# Students' learning style detection using tree augmented naive Bayes

**DOI:** 10.1098/rsos.172108

**Published:** 2018-07-25

**Authors:** Ling Xiao Li, Siti Soraya Abdul Rahman

**Affiliations:** Department of Artificial Intelligence, Faculty of Computer Science and Information Technology, University of Malaya, Kuala Lumpur, Malaysia

**Keywords:** learning styles, Bayesian network, automatic detection

## Abstract

Students are characterized according to their own distinct learning styles. Discovering students' learning style is significant in the educational system in order to provide adaptivity. Past researches have proposed various approaches to detect the students’ learning styles. Among all, the Bayesian network has emerged as a widely used method to automatically detect students' learning styles. On the other hand, tree augmented naive Bayesian network has the ability to improve the naive Bayesian network in terms of better classification accuracy. In this paper, we evaluate the performance of the tree augmented naive Bayesian in automatically detecting students’ learning style in the online learning environment. The experimental results are promising as the tree augmented naive Bayes network is shown to achieve higher detection accuracy when compared to the Bayesian network.

## 1. Introduction

Learning styles are a set of cognitive, emotional, characteristic and physiological factors that serve as the relatively stable indicators of how a student perceives, interacts with and responds to the learning environment [1]. For instance, most of the studies agree on the influence of learning style on the learning attitude, satisfaction level and academic achievement of students in an online education environment, and that the learning style can significantly affect the learning attitude in the education environment. By contrast, when students' learning styles are mismatched, the learning effectiveness is reduced [2]. Past researchers have argued for consideration of learning styles in the development of e-learning systems. This is to maintain students' motivation so that they could learn more effectively [3,4].

To detect learning style, most of the existing approaches can be divided into two types of detection methods: (i) static detection based on the learning style inventory and (ii) dynamic detection through the learning behaviour. Although the static detection method is simple, the lengthy questionnaire on learning style often requires students to be patient in order to complete these questions, and so it often results in poor accuracy measurement and difficult to be dynamically updated [2]. Furthermore, many studies argued that learning style could vary over time and be dependent on task/learning content [5–8]. In order to solve these problems, numerous adaptive learning systems that use dynamic detection method have been developed by using the students’ learning behaviours. Several techniques have been proposed for detecting the learning behaviours such as neural networks, Bayesian networks, rule-based reasoning [9].

Many researchers have validated the effectiveness of the Bayesian network-based automatic detection method. In Feldman's review [9], Bayesian networks are some of the most widely used detection techniques to automatically detect students' learning styles [10–13]. Feldman *et al.* [9] reported the reasons for using the Bayesian network in terms of its natural representation of probabilistic information, and its ability to encode expert knowledge. However, due to some characteristics of the Bayesian network, it leads to the Bayesian network structure becoming unreasonable and affecting the classification accuracy [5].

Besides, when dynamic model is used to detect students’ learning style, a ‘cold start’ problem exists inevitably [14], because (i) there is not enough information available to build users' profile [15] and (ii) the system is unable to infer anything for the new users. To tackle this issue, [10,16] proposed hybrid detection method that combines the static and dynamic methods. As an example, Liyanage *et al.* [10] used the Felder–Silverman learning style model as the basis to predict the learning style during the early stage and followed by the Bayesian network to mine the learning behaviour pattern in order to detect the learning style more accurately. In another approach, Petchboonmee *et al.* [16] used J48 and naive Bayes algorithm to establish the decision tree for the learning style prediction model, which is based on the Kolb learning style model. Although hybrid detection methods have been successful, we argue that simply combining the two detection methods (static and dynamic) remains a major drawback as students are still required to fill out a lengthy questionnaire.

In this paper, we propose a new approach to represent and detect students’ learning style using tree augmented naive Bayesian network to improve the precision of learning style detection. The detection process starts with preset learning styles. Then, it amends and updates the learning style according to the tree augmented naive Bayesian. Experimental results showed the effectiveness of the proposed method.

This paper is organized as follows. Section 2 briefly describes different learning styles that we are considering in this paper. Section 3 presents how the tree augmented naive Bayesian is used in comparison to the Bayesian network. Section 4 describes the detection of learning styles using the tree augmented naive Bayesian, as well as the experimental analysis and results. Section 5 discusses the experimental findings. Finally, §6 concludes the paper and provides direction for future work.

## 2. Learning styles revisited

Generally, a learning style model classifies students according to where they fit in a number of scales that identify the ways in which they receive and process information [17].

In this paper, we consider Felder–Silverman learning style model (FSLSM), because of the main reasons as follows:

(1) FSLSM is the most widely used learning style model. Shockley & Russell [3] analysed the use of learning style model in the adaptive learning system over the past decade and found that the usage amount of FSLSM model ranks the first (50%), much higher than the second Kolb's model (8.6%). The findings of this study are also consistent with those of Akbulut & Cardak [18].
(2) FSLSM provides more detailed descriptions than other learning style models while its reliability and accuracy have also been proven [19].
(3) FSLSM provides a high operational index of learning style (ILS) instrument, which includes 44 questions: 11 questions for each dimension, where each question has two answers to be chosen from, in order to detect both the preference and the degree of preference.

FSLSM divides the learning styles into four dimensions: (i) procession (active/reflective), (ii) perception (sensing/intuitive), (iii) input (visual/verbal) and (iv) understanding (sequential/global) [20].

Procession: active students do not learn much in situations that require them to be passive. Whereas, reflective students do not learn much in situations that provide no opportunity to think about the information being presented. Active students work well in groups; reflective students work better by themselves or at most, with one other person.

Perception: sensing students prefer facts, data and experimentation; whereas intuitive students prefer principles and theories. Sensing students are patient with detail but do not like complications; whereas intuitive students are bored by details and welcome complications.

Input: visual students remember best what they see and this includes pictures, diagrams, time lines, films, and demonstrations. Verbal students remember much of what they hear or read and say.

Understanding: sequential students follow linear reasoning processes when solving problems. Global students make intuitive leaps and may be unable to explain how they come up with solutions. Sequential students can work with the material when they understand it partially or superficially, while global students may have great difficulty in doing so.

## 3. Overview of tree augmented naive Bayesian network

### 3.1. Bayesian network and naive Bayesian network

Bayesian network is an uncertain relationship representation and reasoning model based on probability analysis and graph theory. It is a directed acyclic graph where nodes represent random variables and arcs represent the probabilistic correlation between variables [21].

The absence of edges in a Bayesian network denotes statements of independence. A Bayesian network encodes the following statement of independence about each random variable: a variable is independent of its non-descendants in the network given the state of its parents [22]. A Bayesian network also represents a particular probability distribution, the joint distribution over all the variables represented by nodes in the graph. This distribution is specified by a set of conditional probability tables (CPT). Each node has an associated CPT that specifies this quantitative probability information. Such table specifies the probability of each possible state of the node given each possible combination of states of its parents. For nodes without parents, probabilities are not conditioned on other nodes. These are called the prior probabilities of these variables.

**Figure 1.**
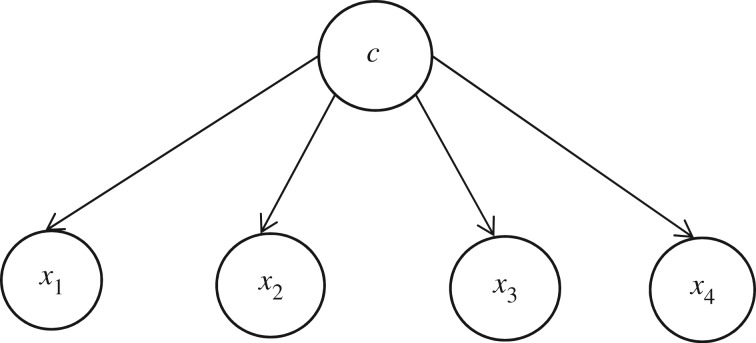
A simple naive Bayesian structure.

Past studies [8] found that Bayesian networks are very suitable to be employed as the detection technique for adaptive education domain. However, the general Bayesian network is too complex for small datasets and easy to overfit [5]. Naive Bayesian avoids this problem. The naive Bayesian classifier is an effective classifier due to two advantages that it has over other classifiers. Firstly, it is easy to be constructed; as the structure is given a priority besides no structure learning procedure is required. Secondly, the classification process is very efficient. Both advantages are derived by assuming that all features are independent of each other. The simple structure only contains two layers, the classification node as the parent node of all other nodes. No other connection is allowed in the naive Bayesian network as shown in figure 1. The only connections link between node c and all leaf nodes *x*_1_, *x*_2_, *x*_3_, *x*_4_; the naive Bayes assumes that all leaf nodes are conditionally independent. But, the conditional independence assumption in the naive Bayes is rarely true in reality. In an adaptive educational domain, the naive Bayesian assumption is (nearly) always violated because the variables are often interconnected.

### 3.2. Tree augmented naive Bayesian network

**Figure 2.**
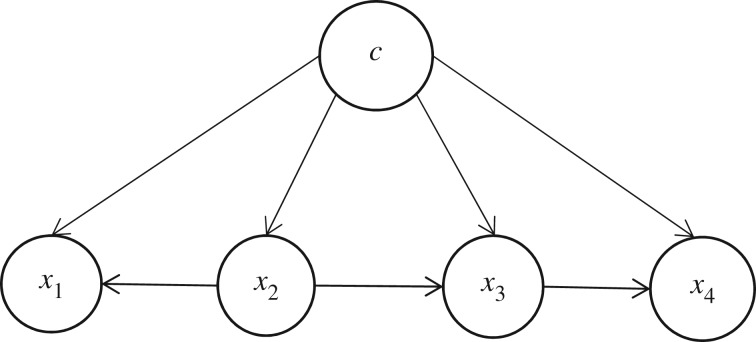
A simple tree augmented naive Bayesian structure.

The requirement that each node must be independent renders the naive Bayesian network structure unreasonable, resulting in the poor accuracy of the naive Bayesian classifier. Friedman *et al*. [23] studied tree augmented naive Bayesian, which extends the naive Bayesian by allowing tree-like structures to be used to represent the dependencies among attributes. Figure 2 shows node c and all leaf nodes *x*_1_, *x*_2_, *x*_3_, *x*_4_ with their respective arcs from node c, from a tree [24]. Tree augmented naive Bayesian makes a good compromise between the general Bayesian network and naive Bayesian. Also, the structure of tree augmented naive Bayesian is simple enough to avoid overfit and strong dependencies can be taken into account.

Unlike naive Bayesian networks, tree augmented naive requires a learning procedure that constructs the model structure. At present, the typical tree augmented naive learning procedure to construct the tree augmented naive classifier uses conditional mutual information.

The algorithm for learning tree augmented naive models is a variant of the Chow-Liu [25] algorithm that is used to learn tree-structured Bayesian networks. Let *C* represent the class variable and {*X_i_*}*_i_*_=1_^*n*^ be the features (non-class variables). The tree augmented naive learning procedure is as follows:

(1) Compute the conditional mutual information:
  $$I(X_{i};X_{j}\;|\;C)=\sum_{x_{i},x_{j},c}P(x_{i},x_{j},c)\log\frac{P(x_{i},x_{j}\;|\;c)}{P(x_{i}\;|\;cP(x_{j}\;|\;c))}.$$
  According to probability theory and information theory, the mutual information of two random variables is a quantity that measures the mutual dependence of the two random variables. Using the conditional mutual information to test the conditional independence of *I*(*X, Y, Z*), where *P*(*·*) is the empirical distribution, computed from the training data. Intuitively, this quantity represents the gain in information by adding *X_i_* as a parent of *X_j_* given that *C* is already a parent of *X_j_*.
(2) Build a complete undirected graph on the features {*X*_1_, …, *X_n_*}, where the weight of the edge between *X_I_* and *X_j_* is *I*(*X_i_*_;_ *X_j_* | *C*).
(3) Find a maximum weighted spanning tree of the completed undirected graph.
(4) Pick an arbitrary node of the maximum weighted spanning tree as the root and set the direction of all edges to be outward from the root to build a directed graph.
(5) Add a class node and an arc between the class node and attribute node to construct tree augmented naive model.

In the current Bayesian network classifiers, tree augmented naive is considered as a widely accepted Bayesian classifier with wide applicability and good comprehensiveness for performance, efficiency, and space–time complexity.

## 4. Learning style detection model based on tree augmented naive Bayesian network

### 4.1. Preset learning style

**Figure 3.**
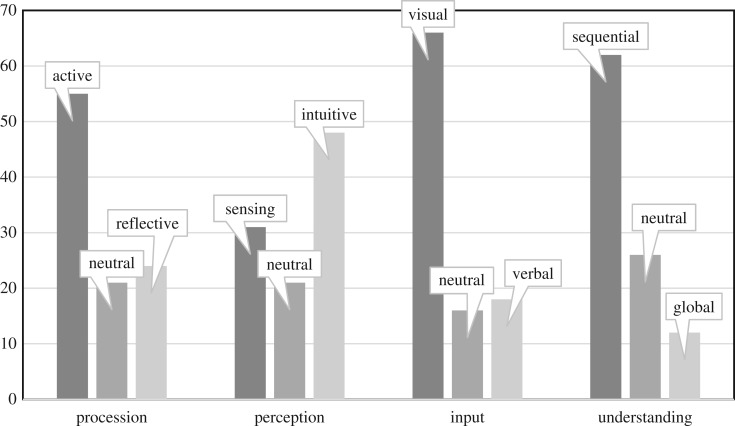
Distribution of the students’ learning styles.

In order to avoid the inconvenience of filling out lengthy learning style questionnaires, this study suggests that the learning style of students be preset at the beginning of learning. The conclusion drawn from the analysis of adult learning style in Shockley and Russell's study [3] found that students are mostly reflective in the information processing dimension, intuitive in the perception dimension, visual for their input, and more sequential in the understanding dimension. In addition, researchers have also studied students' learning style characteristics in different disciplines (biology, commerce, chemistry, finance, accounting, and many more). The results generally prove that the students’ learning styles are characterized by disciplines and specialties. Learning style is also affected by culture, background, different countries [2]. Therefore, in order to identify students' learning style more accurately, this research gathered 46 undergraduates studying bioinformatics. They were required to fill out the ILS instrument online. This requirement was only carried out at the early stage of the experimental study. Figure 3 shows that students’ learning styles in four dimensions are more inclined to the active, intuitive, visual and sequential learning styles. In our study, only the procession dimension is different from Shockley & Russell's result [3], but the other dimensions are the same. In fact, it was discovered that the bioinformatics students required more practical work, while most of the other courses require students to collaborate in a team-based manner. Therefore, the default preset learning styles for these students in the current study are active, intuitive, visual and sequential.

### 4.2. Construction of learning style detection model based on tree augmented naive Bayesian network

**Figure 4.**
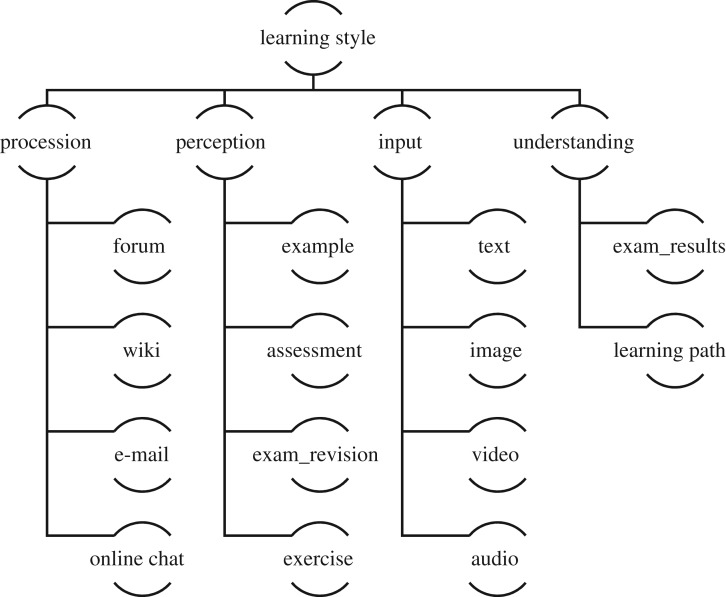
Bayesian network modelling of a student's learning style.

Students enter the system with preset learning styles. Their learning styles will be updated individually based on their learning behaviour as they interact with the system. This learning style detection model is constructed based on tree augmented naive Bayesian network to mine data from students' learning behaviour. The learning behaviours mainly include visiting the forum, sending and receiving e-mail, watching videos, carrying out exercises, communicating, and many more. Based on the literature [12,26,27], an FSLSM-based learning style Bayesian network model was built as shown in figure 4.

Active students work well in groups; reflective students work better by themselves or at most, with one person. Also, we can assess students from their wiki, forums, online chat and e-mail usage to identify if they are active or reflective.

Sensing students prefer facts, data and experimentation whereas intuitive students prefer principles and theories. Sensing students are patient with detail but do not like complications, whereas intuitive students are bored with detail and welcome complications. If a student likes specific learning materials, learns through examples and case studies, prefers to review after exams and carefully examine the questions—these characteristics indicate that he/she is a sensing student. On the other hand, he/she is an intuitive student.

Visual students like to learn using pictures, diagrams, video and animation material. If students like to study using text and audio material, this indicates that they are verbal students.

Sequential students study in a step-by-step manner and follow linear processes according to the learning contents. On the other hand, global students make intuitive leaps and may struggle to explain how they come up with solutions. Additionally, if a student does not read or learn the relevant learning content, but he/she is able to complete the test and obtain high marks, it could be inferred that he/she is a global student.

The recommended setting of variables based on literature [19,28] is presented in table 1. The table describes the different states of the independent variables related to students’ learning behaviour.

**Table 1:** Recommended setting of variables.

dimension	features	description of behaviour
procession	forum	posts messages; replies messages; reads messages; never use
	wiki	very frequently use; occasionally use; never use
	e-mail	very frequently use; occasionally use; never use
	online chat	very frequently use; occasionally use; never use
perception	example	in relation to the number of examples proposed: many (more than 75%); few (25–75%); none
	assessment	in relation to the number of assessments proposed: more than 75%; few (25–75%); none
	exam_revision	in relation to the time assigned to the exam: more than 20%; 10–20%; less than 10%
	exercise	in relation to the number of exercises proposed: many (more than 75%); few (25–75%); none
input	text	in relation to the text-based learning objects proposed: many (more than 75%); few (25–75%); none
	image	in relation to the image-based learning objects proposed: many (more than 75%); few (25–75%); none
	video	in relation to the video-based learning objects proposed: many (more than 75%); few (25–75%); none
	audio	in relation to the audio-based learning objects proposed: many (more than 75%); few (25–75%); none
understanding	exam_results	in relation to the time assigned to the exam: more than 20%; 10–20%; less than 10%
	learning path	in fits and starts; sequential

In this paper, we use procession (*Pro*) dimension node to illustrate the model construction and algorithm implementation. There are two classifications of procession (*Pro*) nodes: active (*Pro1*) and reflective (*Pro2*). Wiki, forum, online chat and e-mail are leaf nodes. The degree of usage is according to students' participation as follows:

(1) Wiki (*W*): very frequently, occasionally, never.
(2) Forum (*F*): post, reply, read, never.
(3) Online chat (*C*): very frequently, occasionally, never.
(4) E-mail (*E*): very frequently, occasionally, never.

Table 2 shows the training dataset from the learning system. Rows represent all students as training data, and columns represent all relevant features of procession dimension, the values indicate the students' behaviour and preference, respectively.

**Table 2.** Training dataset of procession dimension.

student	procession	forum	wiki	online chat	e-mail
1	*Pro1*	*F1*	*W1*	*C1*	*E1*
2	*Pro1*	*F1*	*W1*	*C2*	*E1*
3	*Pro1*	*F1*	*W1*	*C1*	*E2*
4	*Pro2*	*F2*	*W1*	*C3*	*E1*
5	*Pro1*	*F2*	*W1*	*C1*	*E1*
6	*Pro1*	*F2*	*W1*	*C2*	*E3*
7	*Pro2*	*F3*	*W1*	*C3*	*E3*
8	*Pro1*	*F3*	*W1*	*C1*	*E1*
9	*Pro2*	*F4*	*W1*	*C3*	*E2*
10	*Pro1*	*F4*	*W2*	*C3*	*E1*
…	…	…	…	…	…
35	*Pro2*	*F4*	*W3*	*C3*	*E2*
36	*Pro2*	*F4*	*W3*	*C3*	*E1*

We can calculate the CPT of node *Pro* according to the conditional probability *P*(*A*/*B*) = *P*(*AB*)/*P*(*B*) (refer to table 3). The CPT of node *Pro* is the prior probability. The prior probabilities of node *W* and *F* are shown in tables 4 and 5, respectively.

**Table 3.** CPT of node *Pro*.

Pro	value
*Pro1*	16/36
*Pro2*	20/36

**Table 4.** CPT of node *W*.

	Pro	
*W*	Pro1	Pro2
1	4/7	1/8
2	2/7	3/8
3	1/7	4/8

**Table 5.** CPT of node *F*.

	Pro	
F	Pro1	Pro2
*F1*	5/7	0
*F2*	1/7	2/8
*F3*	1/7	2/8
*F4*	0	4/8

### 4.3. Detection algorithm based on tree augmented naive Bayesian network

**Figure 5.**
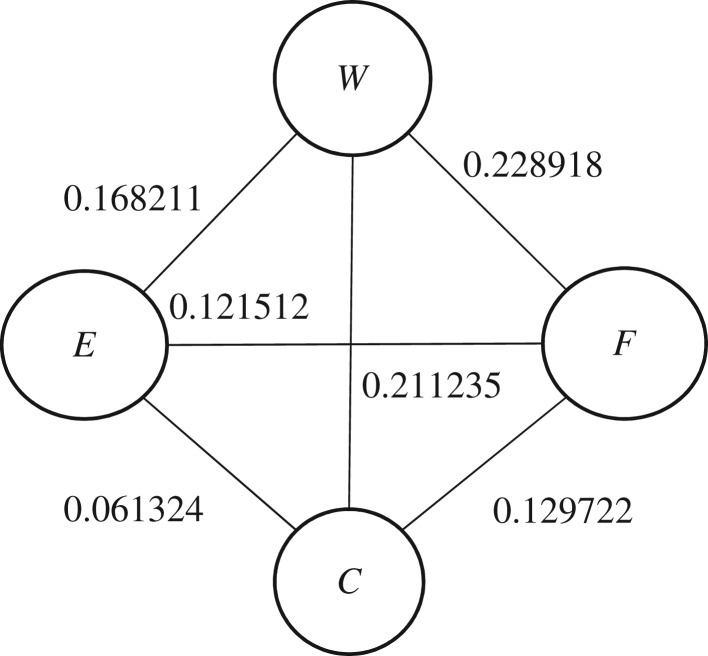
Weighted undirected graph.
  Step 3. Sort the weight *e_i,j_* in descending order, where *e_i,j_* represents weight between the corresponding nodes: *e_w,f_*, *e_f,c_*, *e_w,e_*, *e_w,c_*, *e_f,e_*, *e_c,e_*.
  Step 4. Build the maximum weighed spanning tree: *e_w,f_, e_f,c_, e_w,e_*.
  Step 5. Establish a directed tree using node *C* as the root node and increase the class variable node, the arcs between a class variable node and attribute node. The tree augmented naive Bayesian network structure is established with the class variable as the parent node of all attribute nodes as shown in figure 6.

The steps of learning style detection by tree augmented naive Bayesian network are:

(1) Establish the procession node tree augmented naive Bayesian network structure.
  Step 1. The conditional mutual information between the *W, F, C, E* and *Pro* attribute variables is calculated according to the procedure of tree augmented naive calculation as described above. The results are:
    $$\begin{array}{ll} I_{p}(W;F/Pro)=0.228918 & I_{p}(W;C/Pro)=0.211235\\ I_{p}(W;E/Pro)=0.168211 & I_{p}(F;C/Pro)=0.129722\\ I_{p}(F;E/Pro)=0.121512 & I_{p}(C;E/Pro)=0.061324 \end{array}$$
  Step 2. Construct the weighted undirected graph by weighting the conditional mutual information between pairs of attribute variables as shown in figure 5.

**Figure 6.**
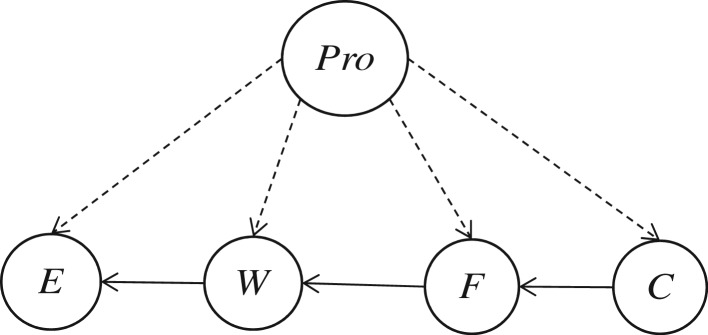
Tree augmented naive network structure.
(2) Establish the tree augmented naive Bayesian network parameters.
  According to the learning process parameter above, CPTs of nodes *C, F, W* and *E* can be calculated separately as shown in tables 6–9.
Tree augmented naive Bayesian network reasoning.
  Assume a given student's learning behaviour set is frequent access to wiki, reading posts, occasional chatting online, occasional e-mailing (*X* = {*W*1, *F*3, *C*2, *E*2}). Respectively, *P*(*X*|*Y_i_*)*P* (*Y_i_*), *i* = 1, 2. The prior probability *P*(*Pro_i_*) for each class can be calculated from the training data: *P(Pro* = ‘active’) = 16/36, *P*(*Pro* = ‘reflective’) = 20/36. The prior probability can be derived as follows:
  $$\begin{array}{rl} P(X\;|\;Pro1) & =\prod_{i=1}^{4}p(X_{i}\;|\;Pa(X_{i})\\ & =p\left(x_{0}=\frac{\text{W1}}{F3},pro1\right)\times p\left(x_{1}=\frac{\text{F3}}{C1},pro1\right)\times p\left(x_{2}=\frac{\text{C1}}{pro1}\right)\\ & \;\times p\left(x_{2}=\frac{\text{E2}}{W1},pro1\right)=\frac{1}{2}\times \frac{2}{9}\times \frac{8}{16}\times \frac{1}{7}=0.07712. \end{array}$$
  Similarly,
  $$\begin{array}{rl} P(X|Pro2) & =\prod_{i=1}^{4}p(X_{i}|Pa(X_{i})\\ & =p\left(x_{0}=\frac{\text{W1}}{F3},pro2\right)\times p\left(x_{1}=\frac{\text{F3}}{C1},pro2\right)\times p\left(x_{2}=\frac{\text{C1}}{pro2}\right)\\ & \;\times p\left(x_{2}=\frac{\text{E2}}{W1},pro2\right)=\frac{1}{6}\times \frac{3}{11}\times \frac{4}{30}\times \frac{1}{3}=0.00371. \end{array}$$
  Therefore, the preliminary result of the tree augmented naive Bayesian network for *X* is *Pro* = ‘active’. Then:
  $$\begin{array}{r} P(Pro=Pro1)=P(X/Pro1)/(P(X/Pro1)+P(X/Pro2))=0.963=95.8\%\\ P(Pro=Pro2)=P(X/Pro2)/(P(X/Pro1)+P(X/Pro2))=0.037=3.8\% \end{array}$$

**Table 6.** CPT of node *C*.

C	Pro = Pro1	Pro = Pro2
*C1*	8/16	4/20
*C2*	5/16	4/20
*C3*	3/16	12/20

**Table 7.** CPT of node *F*.

	C					
F	C1	C2	C3	C4	C5	C6
	*Pro1*	*Pro1*	*Pro1*	*Pro2*	*Pro2*	*Pro2*
*F1*	3/9	3/5	1/3	0	1/2	2/11
*F2*	2/9	2/5	1/3	0	0	1/11
*F3*	2/9	0	1/3	2/4	1/2	3/11
*F4*	1/9	0	0	2/4	0	5/11

**Table 8.** CPT of node *W*.

	F							
*W*	F1	F2	F3	F4	F1	F2	F3	F4
	*Pro*							
	*Pro1*	*Pro1*	*Pro1*	*Pro1*	*Pro2*	*Pro2*	*Pro2*	*Pro2*
*W1*	3/7	3/6	1/2	1	0	0	1/6	1/8
*W2*	2/7	2/6	1/2	0	2/3	1/2	2/6	3/8
*W3*	2/7	1/6	0	0	1/3	1/2	3/6	4/8

**Table 9.** CPT of node *E*.

	*W*					
*E*	W1	*W2*	*W3*	*W1*	*W1*	*W1*
	*Pro*					
	*Pro1*	*Pro1*	*Pro1*	*Pro2*	*Pro2*	*Pro2*
*E1*	5/7	3/7	1	0	1/8	0
*E2*	1/7	3/7	0	1/3	2/8	4/9
*E3*	1/7	1/7	0	2/3	5/8	5/9

The scales of the index for each dimension's learning style of FSLSM are 1,3,5,7,9,11, where 1 and 3 represent learning styles that are fairly well balanced, 5 and 7 indicate a moderate preference, and 9 and 11 indicate a very strong preference. Therefore, 50–100% is divided into three levels, corresponding to the ILS learning style preference levels. A probability of 50–66.7% indicates fairly well balanced, 66.8–83.4% indicates a moderate preference, 83.5–100% shows a strong preference. According to the above calculation, the results show a strong tendency for the ‘active’ on procession dimension.

### 4.4. Experiment and result analysis

#### 4.4.1. Experimental design

We assessed 46 undergraduate bioinformatics students for 7 weeks on genomic technology topics via an online course using learning management system called Moodle. The students had no prior knowledge of the topics, and they were given same static learning materials. Overall, the online course included 396 well-balanced content objects for different learning style dimensions, covered all necessary learning materials, including examples, exercises, images, video tutorials, and many more. The students' learning behaviours were used to detect the individuals’ learning style for comparative analysis between general Bayesian network and tree augmented naive. The learning style of the students was also obtained using the ILS instrument for the comparative analysis and to validate the preset learning styles concept as mentioned before.

#### 4.4.2. Comparative analysis of experimental results

The results obtained by the Bayesian network, tree augmented naive and ILS instrument of four dimensions are shown in table 10.

**Table 10.** Experimental results.

	procession			perception			input			understanding		
user	ILS	BN	TAN	ILS	BN	TAN	ILS	BN	TAN	ILS	BN	TAN
1	ACT	ACT	ACT	INT	INT	SEN	VIS	VIS	NEU	SEQ	NEU	NEU
2	ACT	ACT	REF	INT	NEU	INT	VEB	VEB	VEB	GLO	GLO	GLO
3	NEU	ACT	NEU	SEN	SEN	SEN	VIS	VEB	VIS	GLO	GLO	GLO
4	ACT	NEU	ACT	NEU	INT	NEU	VIS	VIS	NEU	GLO	SEQ	GLO
5	REF	REF	REF	INT	SEN	INT	VIS	VEB	VIS	NEU	NEU	SEQ
6	ACT	ACT	ACT	SEN	SEN	INT	NEU	NEU	NEU	GLO	GLO	GLO
7	ACT	REF	NEU	INT	INT	INT	VIS	VIS	VIS	SEQ	NEU	SEQ
8	NEU	ACT	NEU	INT	NEU	INT	VIS	VIS	VIS	GLO	GLO	NEU
9	ACT	ACT	REF	INT	INT	NEU	VEB	NEU	NEU	SEQ	SEQ	SEQ
10	REF	ACT	REF	NEU	NEU	NEU	VEB	VIS	VEB	SEQ	GLO	GLO
…												
46	ACT	ACT	ACT	SEN	INT	SEN	VIS	VIS	VIS	GLO	NEU	NEU

The precision of Bayesian network and tree augmented naive model learning style detected results can be calculated by using the following formula [12].

$$\text{precision}=\frac{\sum_{i=1}^{n}\text{Sim}(LS_{\mathrm{ILS}},LS_{\mathrm{BNS}/\mathrm{TANS}})}{n}.$$

The precision values obtained are between [0, 1], where 1 means that the learning style obtained by the Bayesian network or tree augmented naive model is the same as that of to the ILS result; and 0 means that the learning style detected by Bayesian network or tree augmented naive model is completely opposite to the ILS result; and 0.5 if one is neutral and the other represents an extreme value; while *n* is the number of students assessed.

The detected results for the precision of Bayesian network model and tree augmented naive Bayesian model in four dimensions of learning style are as shown in table 11.

**Table 11.** Results comparison.

	precision	
dimensions	BN	TAN
procession	62.5	75.3
perception	67.3	72.8
input	73.2	80.0
understanding	65.2	75.2

## 5. Discussion

The results showed that the tree augmented naive has higher precision than the Bayesian network. This is due to the fact that the tree augmented naive algorithm loosens the conditional independence assumption which is consistent with the reality (the interconnection between variables). Comparing with naive Bayesian, tree augmented naive Bayesian allows the additional edges between the attributes of the network in order to capture correlations among them [29]. Furthermore, each attribute can have augmenting edge which encodes statistical dependencies between attributes; therefore, the joint probability of tree augmented naive Bayesian count on the probabilities conditioned not only on class but also from the attribute of parent node [30]. During students' online learning process, many internal connections existed between learning objects within the same learning style dimension, such as ‘online chat’ often appeared together with ‘forum’ section; when the correlation of the interconnection is higher, the result of tree augmented naive achieves better. On the other hand, the tree augmented naive algorithm takes slightly more time than the Bayesian network. This is because the tree augmented naive needs to build the tree on the basis of Bayesian network tree.

In this study, 36 students' data were used for training the classifier. Nevertheless, when looking at the possible variables, for example, in perception dimension, which has 4 different features, 81 (3^4^) possible different states exist because each feature can have 3 states. Using only 36 students as input data might affect the precision of the detected results. Meanwhile, from another point of view, the precision of the results using the proposed approach could be further improved when running in big dataset environment. Another limitation of the current study is that the results of the experiment were only tested on Moodle platform with a specific subject. The consistency of performance needs to be tested when it runs with different learning management platforms or other online courses. Our future work will involve exploring further the performance in different environments.

## 6. Conclusion

We have evaluated the capability of tree augment naive Bayesian to model and detect students' learning styles. The results obtained are positive. Since the tree augmented naive Bayesian network retains the structural features of naive Bayes and relaxes its independence assumption, we could make classifications with higher accuracy. Experimental results prove that the proposed method is more accurate than the results obtained using the Bayesian network.

Although the experiment only assessed restricted numbers of students, the results obtained provide valuable data about students’ learning behaviours with regards to online courses. These data will be used in future to enhance students' learning style modelling. For future work, the experiment will be carried out on a larger scale in order to validate the results obtained so far and to test the performance consistency.

In summary, provided that we take into account issues on learning style detection, the proposed tree augmented naive Bayesian model enables us to discover students' learning styles in a highly precise manner.

## Supplementary Material

LI LINGXIAO_logfile_ESM.csv

## Data accessibility

Data are included as the electronic supplementary material.
